# Cationic nanoparticles-enabled mouthwash combats precancerous oral mucosal inflammation

**DOI:** 10.1093/rb/rbag011

**Published:** 2026-01-30

**Authors:** Meijun Du, Kunyu Wang, Quanxin Ning, Fangman Chen, Renjie Yang, Suwan Ding, Shiming Zhang, Ziming Huo, Xiaochun Xie, Chuanxu Cheng, Bing Shi, Hanyao Huang, Dan Shao, Yi Li, Kam W Leong

**Affiliations:** State Key Laboratory of Oral Diseases and National Clinical Research Center for Oral Diseases, West China Hospital of Stomatology, Sichuan University, Chengdu, Sichuan 610041, China; Department of Oral and Maxillofacial Surgery, West China Hospital of Stomatology, Sichuan University, Chengdu, Sichuan 610041, China; State Key Laboratory of Oral Diseases and National Clinical Research Center for Oral Diseases, West China Hospital of Stomatology, Sichuan University, Chengdu, Sichuan 610041, China; Department of Oral and Maxillofacial Surgery, West China Hospital of Stomatology, Sichuan University, Chengdu, Sichuan 610041, China; State Key Laboratory of Oral Diseases and National Clinical Research Center for Oral Diseases, West China Hospital of Stomatology, Sichuan University, Chengdu, Sichuan 610041, China; National Engineering Research Center for Tissue Restoration and Reconstruction, South China University of Technology, Guangzhou, Guangdong 510006, China; State Key Laboratory of Oral Diseases and National Clinical Research Center for Oral Diseases, West China Hospital of Stomatology, Sichuan University, Chengdu, Sichuan 610041, China; National Engineering Research Center for Tissue Restoration and Reconstruction, South China University of Technology, Guangzhou, Guangdong 510006, China; State Key Laboratory of Oral Diseases and National Clinical Research Center for Oral Diseases, West China Hospital of Stomatology, Sichuan University, Chengdu, Sichuan 610041, China; Eastern Clinic, West China Hospital of Stomatology, Sichuan University, Chengdu, Sichuan 610041, China; Department of Biomedical Engineering, Columbia University, New York, NY 10027, USA; State Key Laboratory of Oral Diseases and National Clinical Research Center for Oral Diseases, West China Hospital of Stomatology, Sichuan University, Chengdu, Sichuan 610041, China; Department of Oral and Maxillofacial Surgery, West China Hospital of Stomatology, Sichuan University, Chengdu, Sichuan 610041, China; National Engineering Research Center for Tissue Restoration and Reconstruction, South China University of Technology, Guangzhou, Guangdong 510006, China; National Engineering Research Center for Tissue Restoration and Reconstruction, South China University of Technology, Guangzhou, Guangdong 510006, China; National Engineering Research Center for Tissue Restoration and Reconstruction, South China University of Technology, Guangzhou, Guangdong 510006, China; State Key Laboratory of Oral Diseases and National Clinical Research Center for Oral Diseases, West China Hospital of Stomatology, Sichuan University, Chengdu, Sichuan 610041, China; Department of Oral and Maxillofacial Surgery, West China Hospital of Stomatology, Sichuan University, Chengdu, Sichuan 610041, China; Department of Biomedical Engineering, Columbia University, New York, NY 10027, USA; State Key Laboratory of Oral Diseases and National Clinical Research Center for Oral Diseases, West China Hospital of Stomatology, Sichuan University, Chengdu, Sichuan 610041, China; Chongqing Key Laboratory of Oral Diseases, Chongqing Municipal Key Laboratory of Oral Biomedical Engineering of Higher Education, Chongqing Municipal Health Commission Key Laboratory of Oral Biomedical Engineering, Department of Oral and Maxillofacial Surgery, The Affiliated Stomatological Hospital of Chongqing Medical University, Chongqing 404100, China; Department of Biomedical Engineering, Columbia University, New York, NY 10027, USA; Department of Systems Biology, Columbia University Medical Center, New York, NY 10032, USA

**Keywords:** oral squamous cell carcinoma, toll-like receptor-9, cell-free DNA, nanoparticle, mouthwash

## Abstract

During the development of oral squamous cell carcinoma (OSCC), multiple danger signals can initiate chronic oral mucosal inflammation, which then gives rise to precancerous and cancerous lesions. Modulation of immune homeostasis is essential to intercept inflammation-driven OSCC. In this study, we aimed to identify key inflammatory danger signals involved in precancerous oral mucosal inflammation and to develop a locally applicable immunomodulatory strategy to prevent this precancerous inflammation. We first identified that saliva cell-free DNA (cfDNA) levels and cfDNA-induced TLR9 activation were linked to OSCC development and progression. Hypothesizing that removing cfDNA would be beneficial for OSCC prevention, we created a cationic nanoparticles-enabled mouthwash that regulates precancerous inflammation via removing negatively charged cfDNA. Both cationic nanoparticles and polymers inhibited the *in vitro* cellular proinflammatory response induced by plasma from OSCC patients and suppressed OSCC patient plasma-induced tumor cell migration and stemness. In the precancerous mouse model, cationic nanoparticles-enabled mouthwash alleviated oral mucosal inflammation via inhibiting TLR9 activation. Overall, our study highlights the role of cfDNA in OSCC progression and the potential of cationic nanoparticle-enabled mouthwash for treating OSCC-related precancerous oral mucosal inflammation.

## Graphical abstract

**Figure:**
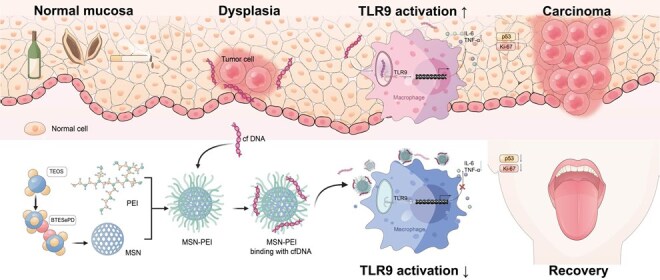

## Introduction

Oral squamous cell carcinoma (OSCC), a common and aggressive head and neck malignancy with a 50% 5-year survival rate and high recurrence, can arise from oral potentially malignant disorders, such as precancerous lesions like oral lichen planus and leukoplakia [1–4]. These lesions and conditions are linked to proinflammatory risk factors like tobacco and alcohol, correlating with chronic oral mucosal inflammation. Additionally, inappropriate inflammation is closely linked to metastasis and cancer progression [5].

In carcinogenesis, the inflammatory microenvironment, regulated by immune cells, plays a significant role [6]. Proinflammatory immune response to both pathogen- and damage-associated molecular patterns (PAMPs and DAMPs) is pivotal in this process [7]. Cell-free DNA (cfDNA) includes nuclear and mitochondrial DNA released from damaged host cells, as well as microbial DNA originating externally, forms a significant portion of PAMPs and DAMPs [8, 9]. These molecules can be detected by toll-like receptor 9 (TLR9) in immune cells, consequently leading to the release of proinflammatory cytokines [10–12]. Although the role of TLR9 and cfDNA-assisted genomic profiling in OSCC is well-studied [13–21], the correlation between cfDNA and OSCC and the exact contribution of cfDNA-TLR9 to OSCC development require further clarification. In this study, we comprehensively establish the potential correlation between cfDNA and OSCC progression in human samples, which suggests that targeting cfDNA-TLR9 signaling pathways could be a promising strategy for controlling precancerous oral mucosal inflammation.

Current clinical approaches for targeting oral mucosal inflammation and treating both precancerous and cancerous lesions include removing dental material irritation, drug therapy and surgical excision, which can be inconvenient for patients and may significantly reduce their quality of life [22]. Given the convenience of mouthwash therapy and the therapeutical potential of targeting cfDNA-TLR9 signaling pathways, we propose enhancing mouthwash therapy with an anti-inflammatory function that targets cfDNA [23]. Cationic biomaterials, especially nanoparticles, have shown therapeutic effects in alleviating inflammatory diseases by removing negatively charged cfDNA [24–30]. Among these nanoparticles, polyethylenimine-functionalized diselenide-bridged mesoporous silica nanoparticles (MSN-PEI) show significant potential for enhancing the mouthwash, with promising prospects for translation into practical therapeutic applications. In this study, we investigate the therapeutic effect of the MSN-PEI-based mouthwash in a mouse model with precancerous oral mucosal inflammation. This nanoparticles-based mouthwash, which removes cfDNA during rinsing and mitigates TLR9 signaling pathway-mediated inflammation, shows notable therapeutic benefits in combating precancerous oral mucosal inflammation and preventing OSCC.

## Materials and methods

Experimental details, including materials, instrumentation, cells, extraction and quantification of cfDNA, enzyme-linked immunosorbent assay (ELISA), mechanical sensitivity (Von Frey) test, synthesis of MSN and MSN-PEI, characterization of MSN-PEI and statistical analysis are listed in supporting information.

### Patient samples

Patient samples (saliva, plasma and tissue samples) were collected from 30 individuals who underwent surgery at the West China Hospital of Stomatology, Sichuan University. Tumor tissues from primary OSCC foci, as well as adjacent non-cancerous tissues, were obtained (5 × 5 × 5 mm³), weighed and immediately processed. These tissues were rinsed with ice-cold PBS to minimize blood contamination, then, dissected into small fragments (1–3 mm³) using a scalpel. Pathological reports were used to confirm the diagnosis of OSCC for all samples. The collection of these samples was conducted following the approval of the Ethical Committee of the West China Hospital of Stomatology, Sichuan University (WCHSIRB-D-2020-461 and WCHSIRB-D-2021-512) and with the informed consent of the patients involved. Demographic information of patients was recorded (Table S1). The collection of patient samples was approved by the Ethical Committee of West China Hospital of Stomatology, Sichuan University (WCHSIRB-D-2020-461 and WCHSIRB-D-2021-512) and written informed consent was obtained from all participants.

### Animal studies

All the mice were bought from Dashuo Biological Technology Company, Chengdu, China. They were 4-week-old male mice on a C57BL/6J background maintained in a sterile room with freely accessible food and randomly allocated to experimental groups. 4-nitroquinoline-1-oxide (4NQO) (50 μg/mL) was used to induce precancerous oral mucosal inflammation in mice [31]. The induction regimen and sampling window were intentionally set to confine lesions to the preneoplastic stage for mechanistic analysis of early inflammatory regulation by the mouthwash, rather than to generate overt OSCC. They were divided into five groups, as the control, PBS + 4NQO, PEI + 4NQO (PEI, 1 mg/mL), MSN + 4NQO (MSN, 10 mg/mL) and MSN-PEI + 4NQO (MSN-PEI, 10 mg/mL, determined by pilot study) groups. 4NQO (50 μg/mL) was delivered into drinking water, for rinsing in the treatment groups, the respective formulation was provided in the drinking water at the indicated concentration, enabling repeated oral mucosal exposure during voluntary drinking, without anesthesia, gavage or manual oral cavity lavage. For the treatment groups, PEI, MSN or MSN-PEI was prepared as an aqueous solution and added into the same-sized mouse drinking-water bottle, with a final volume of 250 mL in each bottle. The bottle was inverted several times to mix thoroughly and ensure uniform distribution of the material in the drinking water, yielding final concentrations of PEI at 1 mg/mL and MSN or MSN-PEI at 10 mg/mL, as described above. Body weight was recorded every week. The mice were sacrificed at 8 weeks post-first-4NQO-exposure and saliva, plasma, tongue tissue and organs (for the toxicity test) were collected. The study procedures were approved by the Ethical Committee of the West China Hospital of Stomatology, Sichuan University (WCHSIRB-D-2024-249).

### Interstitial fluid collection

Tissues were placed in 1.5 mL microcentrifuge tubes and rinsed with chilled PBS. A volume of 100 µL of 0.9% sodium chloride (NaCl) solution was introduced, and the mixture was subsequently centrifuged at a force of 8000 × *g* for 15 minutes at 4°C. The resulting supernatant was carefully collected and preserved at −80°C for subsequent analyses.

### Plasma collection

Blood was collected into an EDTA-containing tube tube and left to stand for 1 h. Afterward, the sample underwent centrifugation at 2000 *g* for a duration of 15 minutes, maintained at a temperature of 4°C. Following this, the plasma layer located at the top was carefully collected and stored at −80°C for further testing.

### Saliva collection

For patients, saliva samples were collected using a saliva collection device on the evening before surgery, after the patients had rinsed their mouths thoroughly with water. All samples were obtained before any surgical procedure or chemoradiotherapy [32]. For mice, the saliva produced by mice during the first 3 minutes following anesthesia was pipetted and gathered. The sample was centrifuged at 2000 *g* for 15 minutes at 4°C. The plasma layer on top was carefully separated and preserved at −80°C for subsequent analysis.

### Histology and immunohistochemistry for human samples

The patient’s tissues were fixed in 4% paraformaldehyde overnight, followed by graded dehydration in 70, 80, 90, 95, 100% ethanol solutions, with 40 minutes of dehydration at each level of ethanol solution. The tissues were immersed in xylene for 3 hours and processed for paraffin embedding. 5-μm paraffin sections were made and dewaxed 3 times with xylene. Then, the sections were dehydrated with gradient ethanol. For immunohistochemistry (IHC) analysis, the expression and location of TLR9 were detected by an anti-TLR9 (1:500), anti-TNF-α (1:200), anti-IL-6 (1:200), anti-Ki67 (1:200) and anti-p53 (1:200). Immunohistochemical (IHC) analysis was performed utilizing an Anti-Rabbit HRP-DAB IHC Detection Kit. Slide scanning was conducted with an Aperio ScanScope slide scanner (Leica Biosystems, Wetzlar, Germany). For quantitative analysis of OSCC and adjacent healthy tissues, the region of interest comprised the epithelial compartment together with the underlying subepithelial connective tissue that contained the inflammatory infiltrate [33] and the integral optical density of positive staining within this region was measured using Image-Pro Plus software (version 6.0.0).

### Histology and immunohistochemistry for mice samples

Mice tissue samples were harvested and frozen in liquid N2-cooled isopentane. Cryosections, each 10 μm thick, were preserved in ice-cold acetone for 10 minutes and stained for histological analysis. Cell slides were fixed by paraformaldehyde for 10 minutes and subsequently stained. For H&E staining, sections were stained using a staining kit. To perform immunohistochemistry (IHC) analysis, an anti-TLR9 (1:500), anti-TNF-α (1:200), anti-IL-6 (1:200), anti-Ki67 (1:200) and anti-p53 (1:200) antibody were used to identify the expression and distribution of TLR9. An Anti-Rabbit HRP-DAB IHC Detection Kit was used for Immunohistochemistry (IHC). Images were captured using a slide scanner from the Aperio ScanScope series, manufactured by Leica Biosystems in Wetzlar, Germany. For quantitative analysis, the integral optical density (IOD) of the positive regions was compared using Image-Pro Plus v6.0.0 software (Media Cybernetics, Rockville, Maryland, USA).

### RNA extraction and quantitative RT-PCR

RNA isolation from cells and tissues was performed using TRIzol reagent, adhering to the supplier’s instructions. The RNA’s concentration and purity were assessed with a NanoDrop One spectrophotometer (Thermo Scientific). cDNA synthesis from the extracted RNA was conducted using the PrimeScript™ FAST RT Reagent Kit with gDNA Eraser. Real-time qPCR was performed with TB Green^®^ Premix Ex Taq™ and primers detailed in Table S2. Relative expression levels were determined using the 2^(−ΔΔCT)^ method.

### DNA binding assay

The interaction between MSN, PEI and MSN-PEI with ct-DNA was assessed following a previously reported method [34]. A 25 µL ct-DNA solution (10 µg/mL), 25 µL PicoGreen and 50 µL MilliQ water were combined in a 96-well plate. The resulting mixture was gently shaken in the dark for 30 minutes to allow complex formation. Subsequently, 100 µL of MSN, PEI or MSN-PEI solutions at varying concentrations were added. The samples were incubated at 37°C for 1 hour, and the fluorescence intensity of the complexes was then measured at 520 nm using a multimode microplate reader (SpectraMax iD3, Molecular Devices), with excitation set to 490 nm.

## Results

### cfDNA levels in body fluids and local cfDNA-TLR9 activation are correlated with the progression of oral squamous cell carcinoma (OSCC)

**Figure 1:**
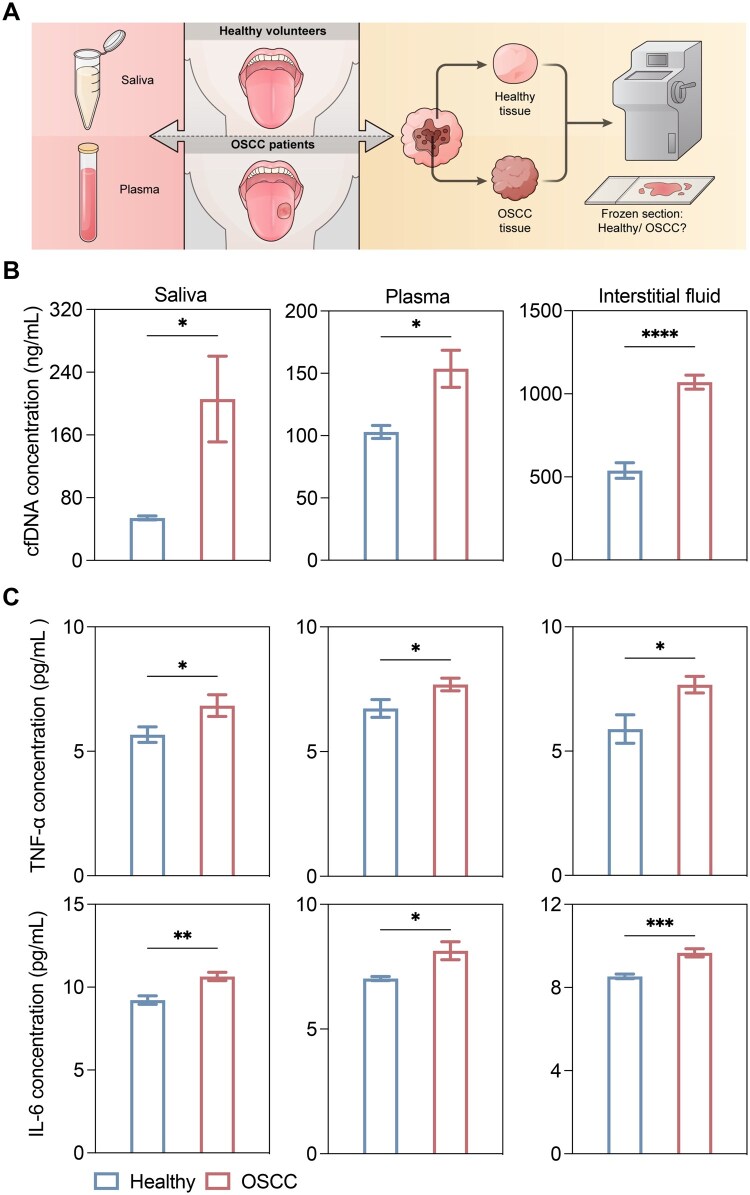
cfDNA, TNF-α and IL-6 levels in body fluids significantly increased in patients with oral squamous cell carcinoma (OSCC). (**A**) Schematic illustration of sample collection. Plasma and saliva samples were collected from healthy volunteers and patients with OSCC. During OSCC resection, tumor tissues and surrounding healthy border tissues were obtained, with tumor and healthy tissues distinguished using frozen sections. (**B** and **C**) cfDNA, TNF-α and IL-6 levels in saliva, plasma and interstitial fluid of healthy volunteers (*n* = 10) and patients with OSCC (*n* = 23). (Data are means ± SEM; *P < 0.05, **P < 0.01, ***P < 0.001, ****P < 0.0001 by two-tailed Student’s *t-*test).

Several studies mentioned that cfDNA can be the potential biomarker for oral cancer [35, 36], but the correlation between cfDNA and OSCC had not been demonstrated previously. Herein, we firstly compared levels of cfDNA and cfDNA-related proinflammatory cytokines in saliva and plasma from healthy volunteers and patients with OSCC, as well as in interstitial fluid between OSCC tissue and the healthy tissue surrounding it (Figure 1A). cfDNA levels in saliva and plasma from patients with OSCC were significantly higher than those in healthy volunteers, and cfDNA levels in interstitial fluid from OSCC tissue were also significantly higher than in the healthy tissue (Figure 1B). The levels of cfDNA-related proinflammatory cytokines, including TNF-α and IL-6, were similarly increased in these body fluids (Figure 1C).

**Figure 2:**
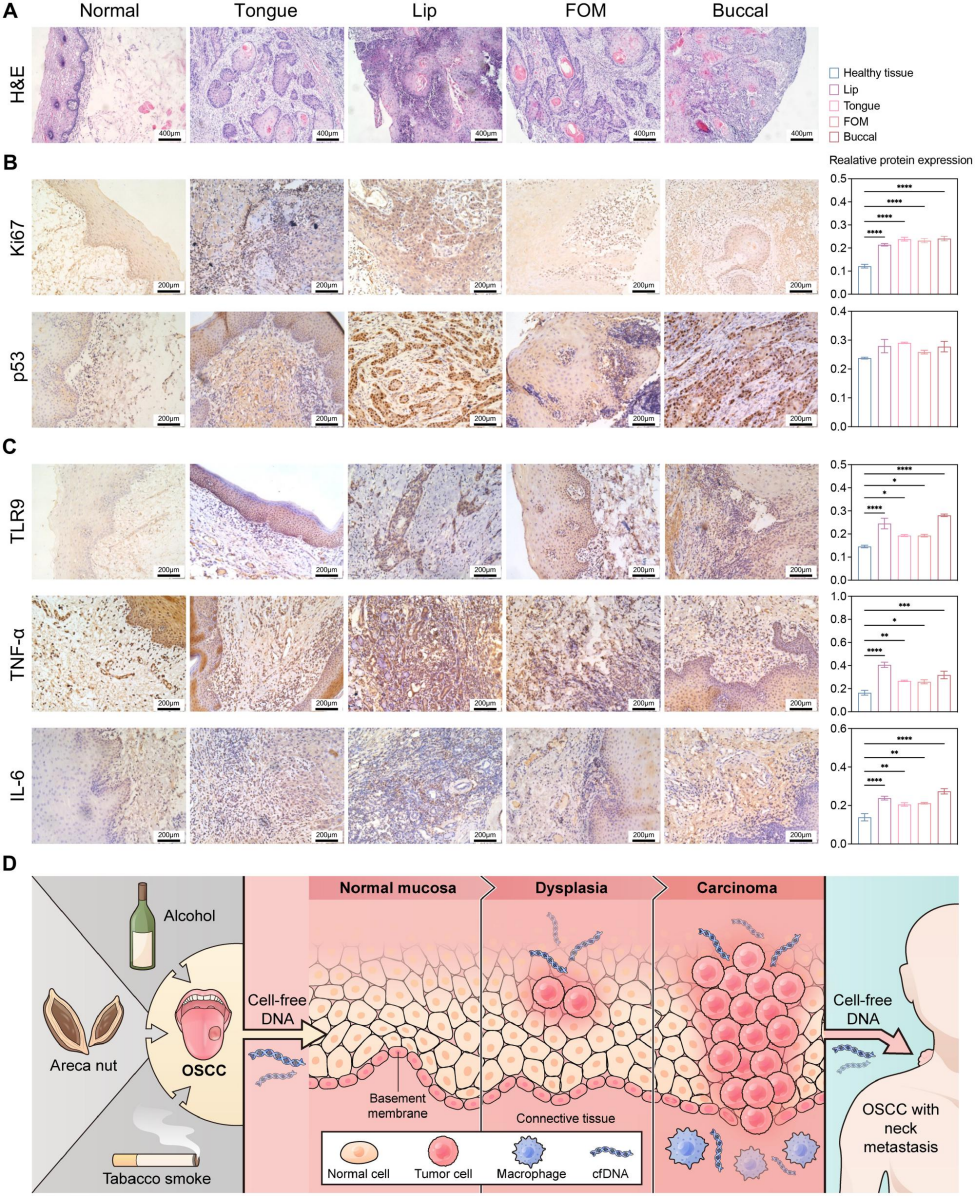
Tumor markers and TLR9-related cytokines expression of OSCC tissues from different sites were significantly higher than in the healthy tissue surrounding the OSCC. (**A**) Representative H&E staining sections of healthy tissue surrounding the OSCC and OSCC tissues from different sites. A denser chronic inflammatory cell infiltrate was also present in the lamina propria, particularly in the subepithelial region, compared with the adjacent healthy mucosa. For quantitative analysis, the region of interest comprised the epithelial compartment together with the underlying subepithelial connective tissue containing the inflammatory infiltrate. The scale bars represent 400 μm. (**B** and **C**) Representative IHC staining sections and expression of Ki67, p53, TLR9, TNF-α and IL-6 in healthy tissue surrounding the OSCC and OSCC tissues from different sites. For quantitative analysis, the region of interest comprised the epithelial compartment together with the underlying subepithelial connective tissue containing the inflammatory infiltrate. The scale bars represent 200 μm. (Data are means ± SEM; *n*  =  3 samples per group; *P  <  0.05, **P  <  0.01, ***P  <  0.001, ****P  <  0.0001 by one-way ANOVA with Tukey’s multiple comparison test). (**D**) Diagram of various irritants such as smoking, alcohol consumption and areca nut chewing, all of which are associated with OSCC. These stimuli can contribute to the destruction of mucosal epithelial cells, leading to the release of circulating free DNA (cfDNA) into the microenvironment. This triggers inflammation in the mucosa, which may progress to dysplasia, carcinoma *in situ*, invasive carcinoma and eventually metastasis.

After confirming the increase in cfDNA, we collected OSCC tissue from different sites of the oral cavity to further investigate TLR9 activation (Figure S1). H&E staining showed that compared to the healthy tissue surrounding the OSCC, OSCC tissues from different sites exhibited obvious infiltrative growth, keratinized bead formation and cellular heterogeneity (Figure 2A). Ki67 and p53 staining revealed significantly higher nuclear Ki67 expression and slightly higher p53 expression in OSCC tissues from different sites of the oral cavity than in healthy tissues, indicating progressive carcinogenesis (Figure 2B). Similarly, while TLR9 was undetectable in the healthy tissues surrounding the OSCC, significantly higher TLR9 expression was observed in OSCC tissues (Figure 2C). TNF-α and IL-6 expressions followed the same pattern as TLR9 (Figure 2C). Additionally, compared to healthy tissues, mRNA levels of *Tlr9*, *Tnf* and *Il6*, as well as mRNA levels of *Arg1*, *Cd86*, *Cd163* and *Cd206* for macrophage phenotype M1 and M2, were all increased in OSCC tissues (Figure S2). Thus, we confirmed that local cfDNA-TLR9 activation and chronic inflammation persist throughout OSCC carcinogenesis (Figure 2D).

We also collected saliva and plasma samples from patients with OSCC metastasis at various stages, as well as bone tissue samples with or without bone invasion. This pilot study indicated an increase in levels of cfDNA present in both saliva and plasma samples collected from patients with lymph node metastasis or higher stage, and an increase in cfDNA-TLR9 downstream signal expression in bone tissue from patients with bone invasion (Figures S3 and S4). However, these results did not reach statistical significance and need to be confirmed in future studies.

### MSN-PEI reduces cellular TLR9-mediated inflammation by removing cfDNA

**Figure 3:**
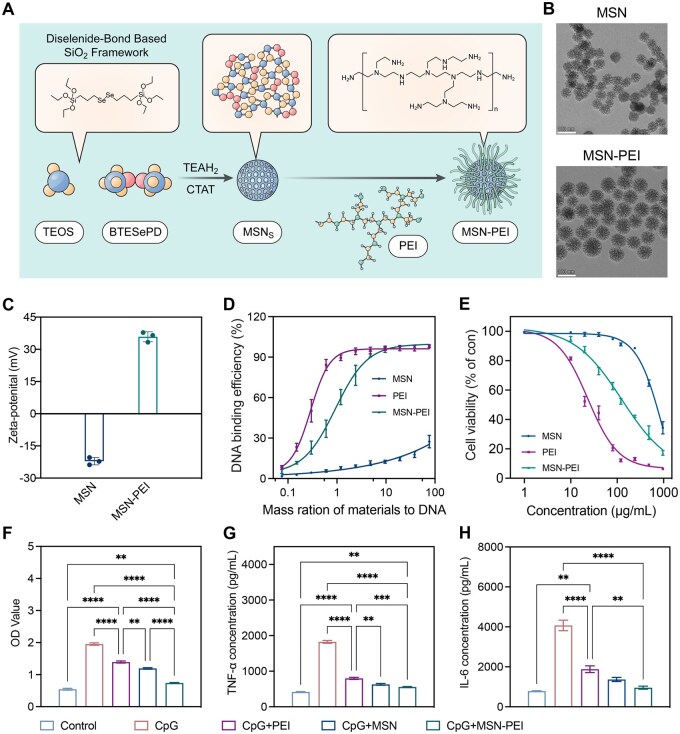
Characterization and anti-inflammatory ability of MSN-PEI *in vitro*. (**A**) Schematic illustration of the fabrication of MSN-PEI by conjugating PEI onto the MSN. (**B**) Representative TEM images of MSN and MSN-PEI. Scale bar, 100 nm. (**C**) Zeta potential of MSN and MSN-PEI. (Data are means ± SEM; *n* = 3 per group.) (**D**) DNA binding efficiency of MSN, PEI and MSN-PEI at different nanoparticle: DNA mass ratios at 37°C. (**E**) Relative fluorescence intensity of CCK-8 in RAW264.7 cells after 24 hours incubation with different with various concentrations of MSN, PEI and MSN-PEI. (*n* = 3 per group. Data are means ± SEM). (**F**) Activation of HEK-TLR9 reporter cells by CpG DNA in the absence or presence of MSN, PEI and MSN-PEI for 24 hours. The corresponding SEAP activity in supernatants from each group is determined with a QUANTI-Blue assay at OD620. (*n* = 3 per group. Data are means ± SEM. **P  <  0.01, ****P < 0.0001 by one-way ANOVA with Tukey’s multiple comparison test.) (**G** and **H**) Inhibitory effects of PEI, MSN and MSN-PEI on CpG-induced secretion of TNF-α and IL-6 by RAW264.7 cells. (*n* = 3 per group. Data are means ± SEM. **P  <  0.01, ***P  <  0.001, ****P < 0.0001 by one-way ANOVA with Tukey’s multiple comparison test.)

Considering the correlation between local cfDNA-TLR9-modulated chronic inflammation and OSCC progression, we opted to use cationic nanoparticles to enhance the mouthwash’s ability to remove cfDNA [24, 25]. Mesoporous silica nanoparticles (MSNs) with diselenide bridges, averaging around 60 nm in diameter, were prepared (Figure 3A and B and Figure S5A). The MSNs featured a pore size distribution centered at 5.26 nm, with a surface area of 550.90 m^2^/g and a pore volume of 0.88 cm³/g (Figure S3B and C). The nanoparticles were functionalized with PEI-25k (20 wt%, as determined by thermogravimetric analysis) to produce MSN-PEI (Figure S6A). This process resulted in a positively charged surface, confirmed using a Zetasizer, while preserving the original morphology and size, as verified by TEM and SEM analyses (Figure 3B and C and Figure S6B). To evaluate formulation stability under relevant pH conditions, MSN-PEI was incubated at pH 6.0 and pH 7.0, and its hydrodynamic size and zeta potential were monitored over 24 hours. Both parameters remained stable across the tested time points, and TEM images showed no obvious aggregation, supporting good colloidal stability of MSN-PEI within this pH range (Figure S6D–F). While MSNs showed no binding affinity for calf thymus DNA (ct-DNA), both free PEI and MSN-PEI exhibited a high binding affinity for ct-DNA (Figure 3D). Importantly, MSN-PEI significantly decreased cytotoxicity of free PEI in RAW 264.7 cells and HEK-Blue TLR9 cells (Figure 3E and Figure S6C). MSN-PEI (100 mg/L) showed degradation when exposed to 100 μM H_2_O_2_ solution at 37°C under constant rotation. Samples were collected for TEM analysis at 24 and 48 hours (Figure S7).

We assessed the ability of MSN-PEI to capture cfDNA and inhibit TLR activation. Both MSN-PEI and free PEI significantly suppressed CpG DNA-induced TLR9 activation, leading to reduced TNF-α as well as IL-6 secretion by macrophages (Figure 3F–H). While unmodified MSNs also inhibited TLR activation and cytokine secretion, their effectiveness was lower compared to PEI and MSN-PEI. Overall, MSN-PEI effectively removed cfDNA, inhibiting cellular TLR9-mediated activation and inflammation *in vitro*.

### cfDNA removal by MSN-PEI affects biological behavior of cancer cells *in vitro*

**Figure 4:**
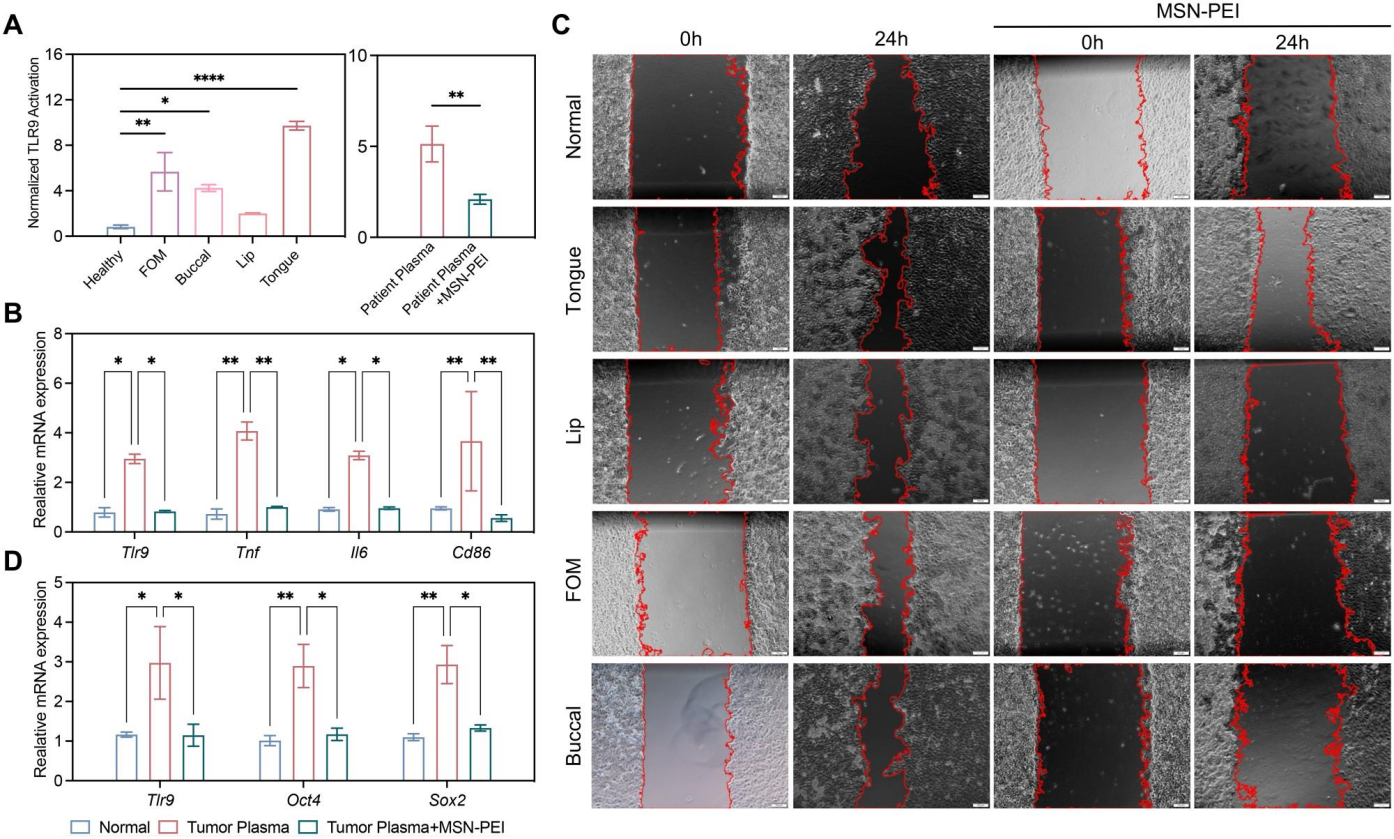
MSN-PEI Affected OSCC-related TLR9 proinflammatory response and biological behavior of cancer cells *in vitro*. (**A**) Activation of HEK-TLR9 reporter cells by plasma from patients with OSCC (different sites) in the absence or presence of MSN-PEI for 24 hours. The corresponding SEAP activity in supernatants from each group is determined with a QUANTI-Blue assay at OD620. (*n* = 3 per group; Data are presented as mean ± SEM; *P < 0.05, **P  <  0.01, ****P < 0.0001 by one-way ANOVA with Dunnett’s multiple comparison test or two-tailed Student’s *t-*test.) (**B**) mRNA expression levels of *Tlr9*, *Tnf, Il6* and *Cd86* in RAW 264.7 cells by plasma from patients with OSCC in the absence or presence of MSN-PEI for 24 hours. (*n* = 3 per group; Data are presented as mean ± SEM; *P < 0.05, **P  <  0.01, ****P < 0.0001 by one-way ANOVA with Dunnett’s multiple comparison test.) (**C**) Migration of CAL 27 cells by activation of plasma from patients with OSCC in the absence or presence of MSN-PEI for 24 hours in the scratch assay. (**D**) mRNA expression levels of *Tlr9, Oct10* and *Sox2* in CAL 27 cells by plasma from patients with OSCC in the absence or presence of MSN-PEI for 24 hours. (*n* = 3 per group; Data are presented as mean ± SEM; *P < 0.05, **P  <  0.01, ****P < 0.0001 by one-way ANOVA with Dunnett’s multiple comparison test.)

Plasma from patients with OSCC located at different sites induced significantly elevated in TLR9 activation in HEK-Blue TLR9 cells compared to that from healthy volunteers, but this activation could be inhibited by MSN-PEI (Figure 4A and B and Figure S8). The increasing expression levels of *Tlr9*, *Tnf, Il6* and *Cd86* in RAW 264.7 induced by the patient plasma suggested significant cellular inflammation, which could be alleviated by MSN-PEI (Figure 4B). Additionally, the levels of proinflammatory cytokines in RAW 264.7 after stimulation with patient plasma were also reduced by MSN-PEI (Figure S9).

CAL 27 cells, epithelial cells derived from a male patient with OSCC [37], were used to study the biological behavior influenced by patient plasma and MSN-PEI. Plasma from patients with OSCC at different sites induced a more pronounced and rapid migration of CAL 27 cells in the scratch assay compared to the control group, which could be inhibited by MSN-PEI treatment (Figure 4C). The increased expression levels of *Tlr9*, *Oct4* and *Sox2* induced by the patient plasma suggested enhanced stemness of CAL 27 cells, which could be controlled by MSN-PEI (Figure 4D). The levels of proinflammatory cytokines in CAL 27 cells after stimulation with patient plasma were also reduced by MSN-PEI (Figure S10).

### MSN-PEI-based mouthwash combats precancerous oral mucosal inflammation by removing cfDNA

**Figure 5:**
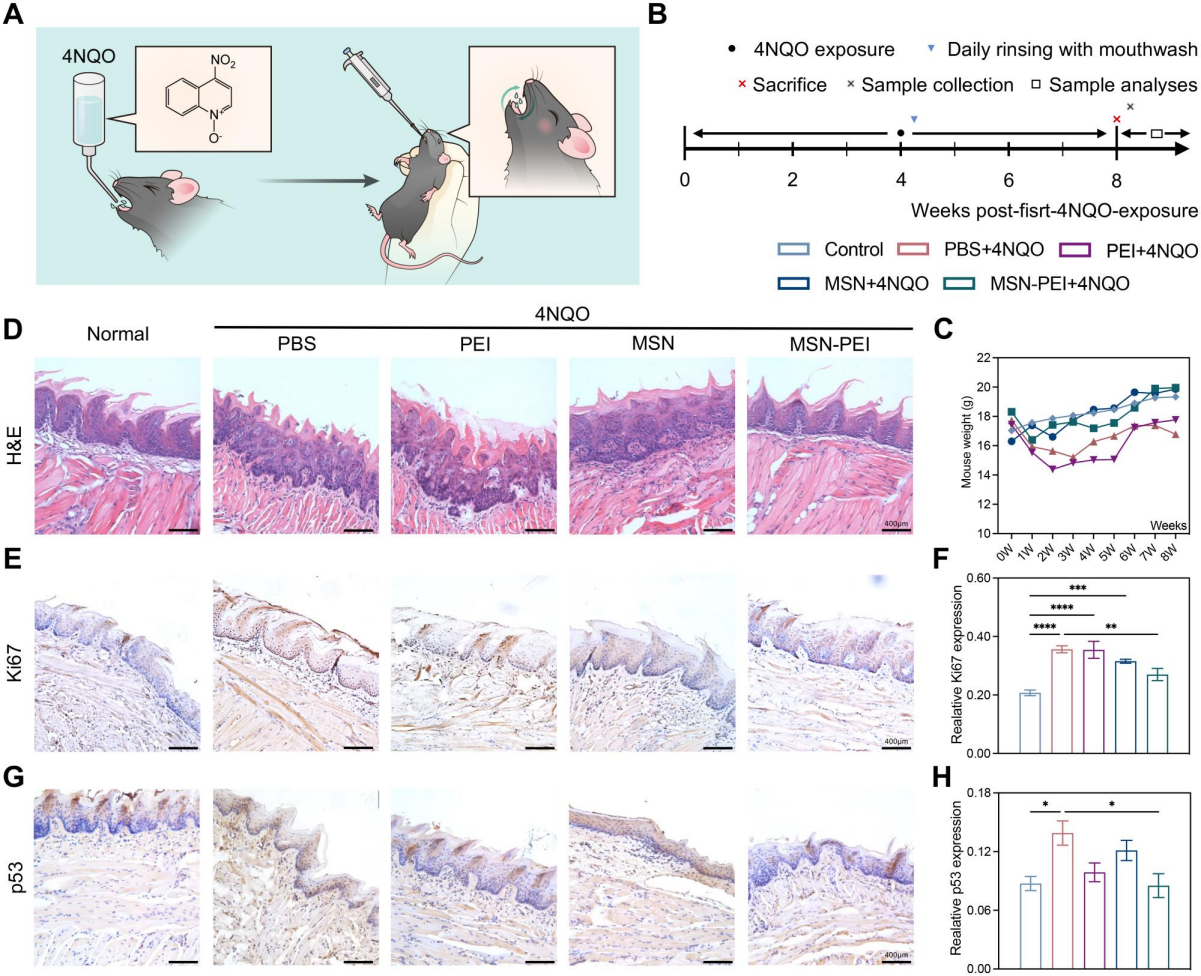
MSN-PEI-based mouthwash inhibits precancerous oral mucosal inflammation. (**A**) Schematic diagram of the animal model with precancerous oral mucosal inflammation by 4NQO stimuli and the application of daily rinsing with mouthwash. 4NQO was added to the drinking water and therapeutic nanomaterials were administered by daily rinsing. (**B**) Experimental schedule. Five groups were designed, as the control, PBS + 4NQO, MSN + 4NQO and MSN-PEI+4NQO groups. (6 mice per group.) (**C**) Weight changes in different groups. (**D**) Representative H&E staining sections of tissue from different groups, with prominent subepithelial inflammatory cell infiltration in the lamina propria. The scale bars represent 400 μm. (**E** and **F**) Representative IHC staining sections and expression of Ki67 in tissue from different groups. The scale bars represent 400 μm. (Data are means ± SEM; *n*  =  3 samples per group; **P  <  0.01, ***P  <  0.001, ****P  <  0.0001 by one-way ANOVA with Tukey’s multiple comparison test). (**G** and **H**) Representative IHC staining sections and expression of p53 in tissue from different groups. The scale bars represent 400 μm. (Data are means ± SEM; *n*  =  3 samples per group; **P  <  0.01 by one-way ANOVA with Tukey’s multiple comparison test).

4-nitroquinoline-1-oxide (4NQO) (50 μg/mL) was used to induce precancerous oral mucosal inflammation in mice [31]. The mouthwash was applied to the mice once per day to simulate daily mouthwash use (Figure 5A and B). The concentration of MSN-PEI in mouthwash, 10 mg/mL, was determined in a pilot study (Figure S11). To confirm the therapeutic effect of MSN-PEI-based mouthwash, five groups were designed, including the control, PBS + 4NQO, PEI + 4NQO, MSN + 4NQO and MSN-PEI + 4NQO groups. After 8 weeks of model establishment and mouthwash treatment, mice treated with MSN-PEI and MSN-based mouthwashes maintained their weight, whereas mice in the PBS + 4NQO and PEI + 4NQO groups experienced a significant weight loss (Figure 5C). Tongue tissues were collected, and H&E staining showed significant heterogeneous hyperplasia and infiltrative growth of the squamous epithelium in the PBS + 4NQO group. This condition was alleviated by the modified mouthwash, with the MSN-PEI-based mouthwash showing the highest efficacy (Figure 5D). Ki67 and p53 staining indicated that expressions of Ki67 and p53 were significantly higher in the PBS + 4NQO group compared to the control group, but these expressions were notably reduced by the MSN-PEI-based mouthwash (Figure 5E–H). Additionally, the MSN-PEI-based mouthwash preserved the mice’s sensitivity to pinch and mechanical stimuli after the induction of precancerous oral mucosal inflammation (Figure S12). In terms of safety, no gross organ damage was detected after mouthwash application (Figure S13).

**Figure 6:**
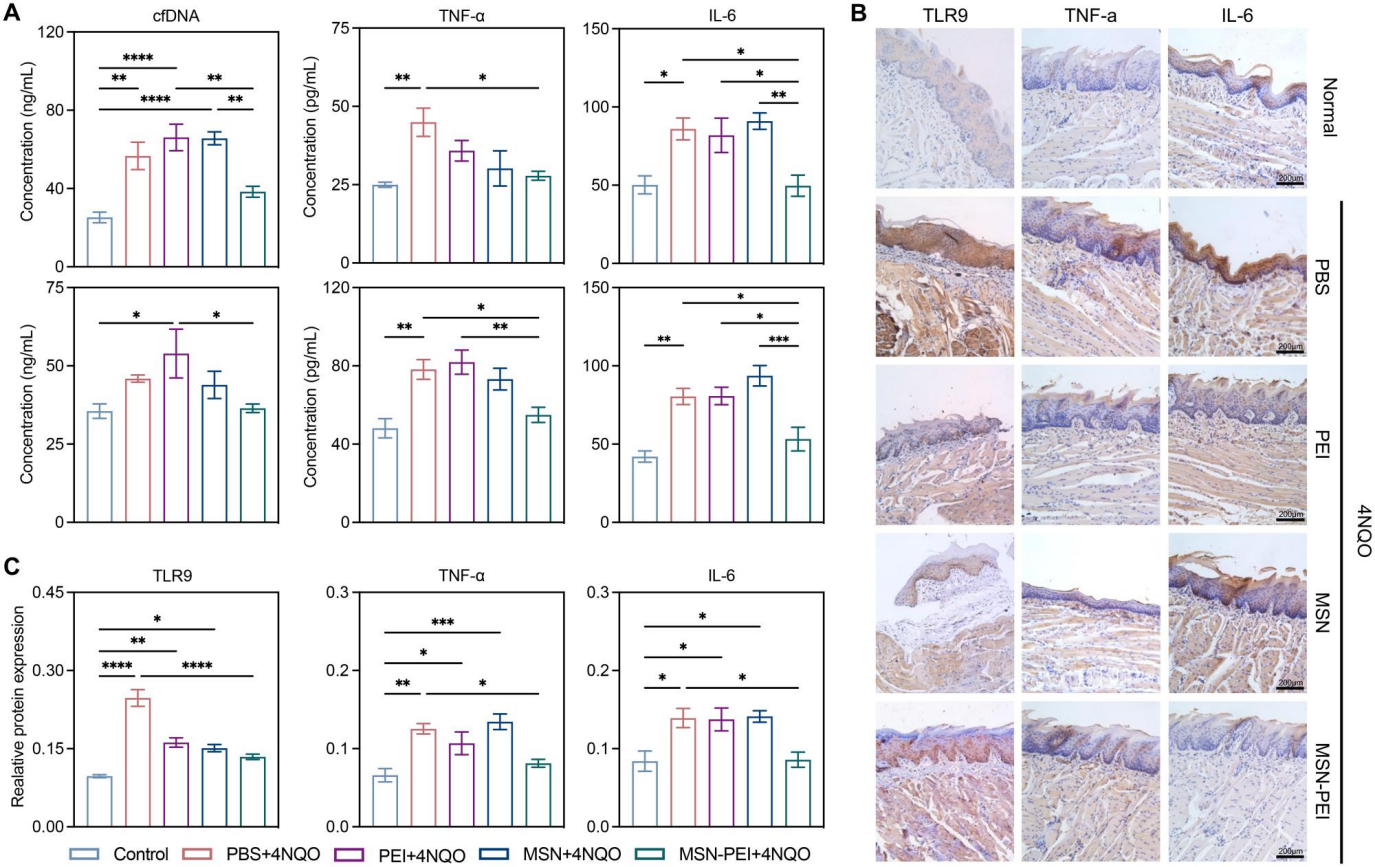
MSN-PEI-based mouthwash combats precancerous oral mucosal inflammation by removing cfDNA. (**A**) cfDNA, TNF-α and IL-6 levels in saliva, plasma and interstitial fluid of the five groups, as the control, PBS + 4NQO, MSN + 4NQO and MSN − PEI + 4NQO groups. (Data are means ± SEM; *P < 0.05, **P < 0.01, ***P < 0.001, ****P < 0.0001 by one-way ANOVA with Tukey’s multiple comparison test). (**B** and **C**) Representative IHC staining sections and expression of TLR9, TNF-α and IL-6 of the tissue from the five groups. The scale bars represent 200 μm. (Data are means ± SEM; *n*  =  3 samples per group; *P  < 0.05, **P  <  0.01, ***P  <  0.001, ****P  <  0.0001 by one-way ANOVA with Tukey’s multiple comparison test).

We then investigated whether this inhibition was mediated by cfDNA removal. Levels of cfDNA, TNF-α and IL-6 in saliva and plasma increased significantly after 4NQO stimulation, but these levels were notably reduced by the MSN-PEI-based mouthwash (Figure 6A). TLR9, TNF-α and IL-6 immunohistochemical staining in tongue tissues demonstrated that MSN-PEI-based mouthwash could significantly alleviate TLR9-mediated inflammation (Figure 6B and C). Thus, MSN-PEI-based mouthwash showed a favorable therapeutic effect in combating precancerous oral mucosal inflammation by removing cfDNA and inhibiting TLR9 activation.

## Discussion

OSCC develops in the mucosal epithelium of the oral cavity through persistent exposure to various risk factors, such as tobacco, alcohol, areca nut and human papillomavirus (HPV) [38]. These factors disrupt the mucosal epithelium’s homeostasis, initiating the development of oral potentially malignant disorders and precancerous lesions [4]. The process of oral malignant transformation involves epithelial hyperplasia, varying degrees of dysplasia, carcinoma *in situ* and ultimately invasive carcinoma, which can result in distant metastasis [39]. Inflammatory infiltrate significantly increases during this transformation process, manifested by various oral inflammatory conditions such as oral lichen planus (OLP) and submucous fibrosis [37]. Additionally, other inflammatory diseases in the oral cavity, such as periodontitis, are associated with carcinogenesis as well [40]. Thus, modulating the local inflammatory microenvironment can be the potential strategy to combat precancerous oral mucosal inflammation and prevent OSCC development and progression.

The inflammatory microenvironment is regulated by immune cells, with TLR9 playing a significant role in modulating their function [6]. cfDNA, which refers to the total DNA shed into body fluids under physiological and pathological conditions, is the key ligand for TLR9 activation and triggers inflammation [25, 26]. cfDNA includes endogenous nuclear and mitochondrial DNA released by damaged host cells, as well as exogenous bacterial or viral DNA [8, 9]. After exposure to risk factors, the oral mucosa microenvironment can accumulate large amounts of cfDNA, which chronically activates TLR9-NF-κB-mediated proinflammation. Our study first demonstrated that cfDNA levels were significantly elevated in the body fluids of patients with OSCC and notably higher in the interstitial fluids of OSCC tissue compared to healthy tissue surrounding the tumor.

This finding further confirms the potential contribution of cfDNA to the inflammatory tumor microenvironment of the oral mucosa, as cfDNA can cause persistent TLR9 activation. Our results showed that OSCC from different sites of the oral cavity have similar significant TLR9 activation than the healthy tissues. Evidence has shown that TLR9-related inflammation can be tumorigenic and promote carcinogenesis [41, 42], with TLR9 playing a key role in the malignant transformation and invasion of OSCC [14, 17, 18, 43]. It is also found that bacterial DNA could influence OSCC aggressiveness via TLR9 activation [44]. In this study, we also preliminarily observed a potential association between cfDNA-TLR9-related proinflammatory responses and lymph node metastasis and bone invasion of OSCC. Together, these observations support a model in which accumulated cfDNA chronically activates TLR9 in both immune and epithelial compartments, thereby shaping the tumor immune microenvironment. In macrophages, sustained TLR9 signaling promotes persistent production of proinflammatory cytokines such as TNF-α and IL-6 and drives polarization toward a tumor-promoting, matrix-remodeling phenotype, which favors malignant progression rather than resolution of inflammation. In parallel, TLR9 engagement enhances NF-κB-associated proliferative and survival signaling and cooperates with genetic instability, as reflected by increased Ki67 and p53 expression. Under this framework, scavenging cfDNA and attenuating TLR9 activation using MSN-PEI is expected to dampen these protumorigenic inflammatory cues, thereby reducing the risk of malignant transformation. However, larger sample sizes are needed to validate these findings.

After confirming that cfDNA-TLR9-related proinflammation is potentially involved in OSCC development and progression, targeting this proinflammatory response by modulating the cfDNA-TLR9 pathway should be a promising strategy. Anti-inflammatory approaches using cationic biomaterials to capture or remove cfDNA have been applied in various inflammatory diseases [24–27, 45–47]. For instance, cationic hydroxyapatite nanoparticles and cationic hydrogels have been shown to inhibit periodontitis by clearing cfDNA [26, 48]. Additionally, cationic nanoparticles have been found to inhibit chemotherapy-induced breast cancer metastasis [27]. To expand the application of cationic biomaterials, this study explores whether they can prevent carcinogenesis by combating local precancerous mucosal inflammation.

MSN features a functionalized surface with high modifiability, along with good retention and biocompatibility [49]. It has been reported that orally administered MSN-PEI can attenuate colonic and peritoneal inflammation and ameliorate colitis [24]. Consequently, MSN-PEI is a promising candidate for treating precancerous oral mucosal inflammation. Our *in vitro* studies confirmed that MSN-PEI can inhibit cellular inflammation induced by body fluids from patients with OSCC and control cancer cells’ migration and stemness. A variety of cfDNA-targeting or TLR9-inhibiting strategies have been explored in oncology and inflammatory diseases, including systemically administered cfDNA-scavenging cationic nanomedicines for sepsis and inflammatory bowel disease, small-molecule or oligonucleotide-based TLR9 antagonists and downstream anti-inflammatory agents that broadly converge on NF-κB signaling pathways [24, 25, 50–52]. While these systemic approaches can effectively suppress inflammation, they typically require higher total doses and are associated with off-target effects, including generalized immunosuppression and increased infection risk. In contrast, our MSN-PEI formulation is designed as a locally applied mouthwash for the oral cavity, enabling high cfDNA-scavenging capacity directly at the diseased mucosal surface while requiring substantially lower overall doses. The cationic nature of MSN-PEI not only facilitates electrostatic capture of negatively charged cfDNA, but also confers mucoadhesive properties that may prolong mucosal residence time and enhance local efficacy. By restricting activity to the site of disease and minimizing systemic exposure, this local nanoparticle-based approach offers a distinct translational advantage for the long-term management of chronic precancerous oral mucosal inflammation.

Current clinical and practical approaches for treating oral mucosal inflammation include using anti-inflammatory mouthwashes, removing sources of dental material irritation, drug therapy and surgical excision [22, 53–56]. Mouthwash is the easiest and most convenient option for patients. We propose enhancing mouthwash therapy by incorporating MSN-PEI for local administration to better control oral mucosal inflammation, and for simulating daily mouthwash use, it was applied to the mice once per day. The animal model with 4NQO-induced precancerous oral mucosal inflammation was chosen to simulate the process of OSCC carcinogenesis, aiming to replicate the long-term exposure of the human oral cavity to stimuli such as smoking and drinking [57]. In clinical practice, an MSN-PEI-based mouthwash would be positioned as an adjunctive, disease-modifying formulation for patients with chronic precancerous oral mucosal inflammation, rather than as a conventional antiseptic rinse. Widely used mouthwashes such as chlorhexidine primarily exert antibacterial effects and are effective for plaque and gingivitis control; however, their long-term use is limited by adverse effects, including tooth staining, taste disturbance and potential disruption of the oral microbiota [58–60]. In contrast, the MSN-PEI mouthwash is designed as a locally acting cfDNA-scavenging rinse with a short mucosal contact time and minimal systemic exposure, aiming to attenuate inflammation by targeting an upstream inflammatory driver rather than broadly suppressing microbial load.

MSN-PEI-based mouthwash demonstrated a significantly better therapeutic effect in combating precancerous oral mucosal inflammation and preventing OSCC development compared to free PEI-based mouthwash. This improvement may be attributed to the nanoparticles, which help the cationic biomaterials stay in the mucus, thereby enhancing their ability to remove cfDNA. MSN-PEI-based mouthwash can stably maintain the levels of cfDNA, TNF-α and IL-6 in saliva and plasma and inhibit TLR9 activation in tissue, indicating that cationic mouthwash can modulate the local mucosal microenvironment. From a translational perspective, this topical formulation is suitable for repeated administration during a preneoplastic risk window, either as a maintenance regimen integrated into daily oral care or as a short-intensified course during episodes of mucosal inflammatory exacerbation. Our study also preliminarily demonstrated that cationic biomaterials can potentially control cancer pain [61]. Together, cationic nanoparticles-based mouthwash is a promising and translatable approach for combating precancerous and other oral mucosal inflammation and the optimal frequency and duration, together with long-term biosafety and potential effects on oral microbiota and mucosal barrier integrity, warrant prospective clinical evaluation.

As our study demonstrated that cationic nanoparticles-based mouthwash exhibited promising therapeutic efficacy in oral mucosal inflammation, there are still some limitations to consider. The mucus, being the most direct body fluid in contact with the mucosa, should be tested to understand the local microenvironment. However, collecting mucus from the oral mucosa of mice presents significant challenges. Moreover, further investigations are necessary to validate the findings of this study. This includes studying the effect and mechanism of cationic nanoparticles-based mouthwash on cell behavior involved in OSCC carcinogenesis *in vivo*, as well as assessing the efficacy of the mouthwash in other experimental precancerous and OSCC models. In addition, the long-term biosafety of repeated oral exposure to MSN-PEI requires dedicated assessment in chronic-use studies, even though mesoporous silica nanoparticles have demonstrated favorable safety and tolerability profiles in oral administration and clinical trials [33, 62]. Potential effects on oral microbiota composition, mucosal barrier integrity and local immune homeostasis should be systematically evaluated in the future. From a regulatory standpoint, future development will need to address product classification, manufacturing quality control and compliance with regulatory standards for topical oral formulations prior to clinical testing.

## Conclusion

**Figure 7:**
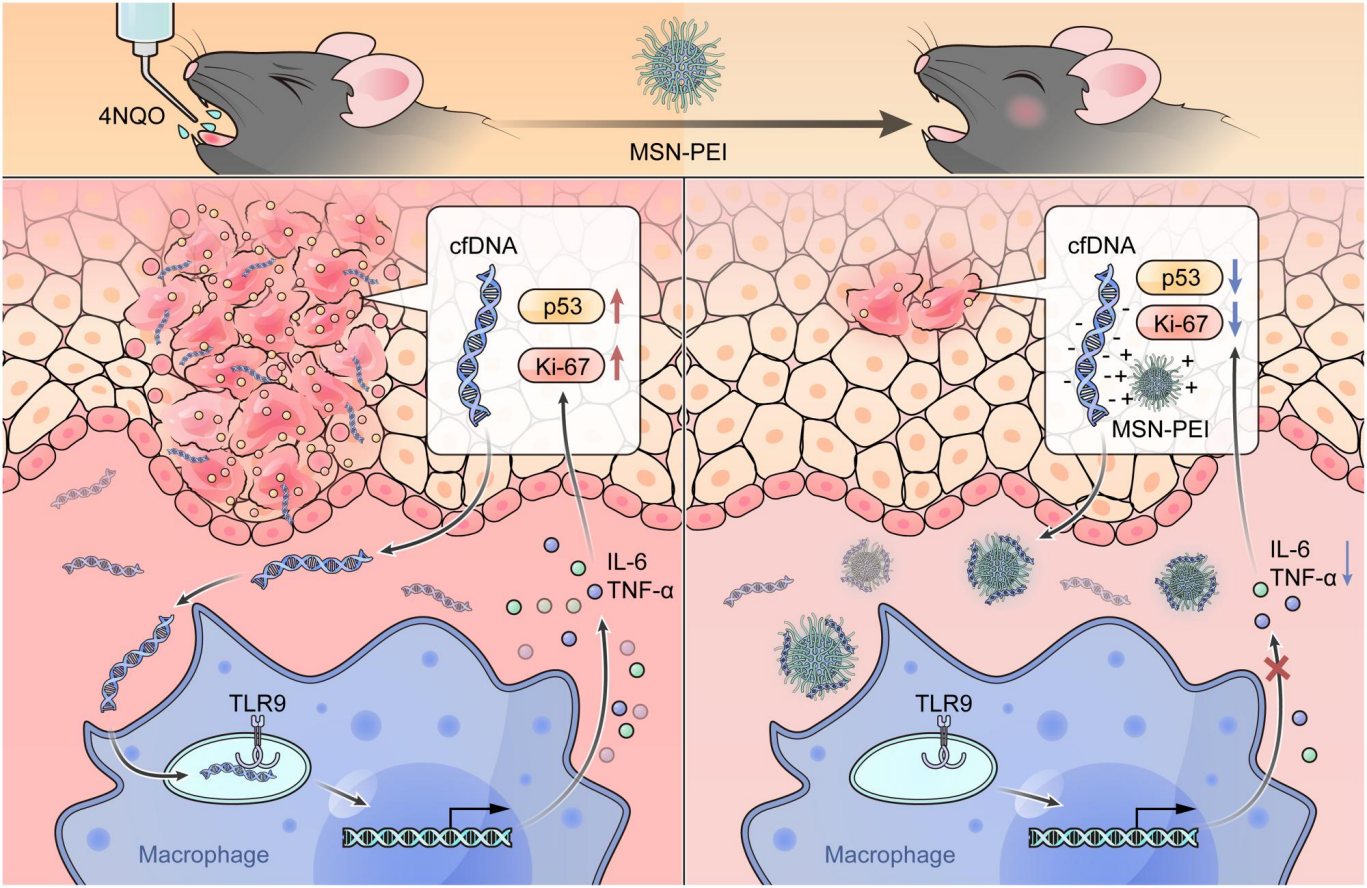
Schematic illustration of how nanoparticle-based mouthwash combats precancerous oral mucosal inflammation.

Our study demonstrated that cfDNA levels and cfDNA-induced TLR9 activation were associated with OSCC development and progression (Figure 7). Cationic nanoparticles-based mouthwash offers a promising approach to alleviate precancerous oral mucosal inflammation by removing cfDNA in the local microenvironment.

Persistent and chronic oral mucosal inflammation is induced by 4NQO, triggering the release of cfDNA, activation of TLR9 and secretion of proinflammatory cytokines such as TNF-α and IL-6, ultimately leading to the development of precancerous lesions. Daily rinsing with MSN-PEI-based mouthwash can reduce inflammatory cytokines and inhibit the development of precancerous lesions by absorbing and removing cfDNA.

## Supplementary Material

rbag011_Supplementary_Data

## Data Availability

The data supporting this study’s findings are available from the corresponding author upon reasonable request.
