# Supplementary material for: Cationic nanoparticles-enabled mouthwash combats precancerous oral mucosal inflammation
Source: Regen Biomater. 2026 Jan 30;13:rbag011. doi: 10.1093/rb/rbag011 (PMC12962229; doi:10.1093/rb/rbag011)
Supplement: rbag011_Supplementary_Data [file rbag011_supplementary_data.pdf]

Supporting Information

Supplemental Materials and Methods

Materials

The Gibco company (Carlsbad, CA, U.S.A.) supplied Dulbecco's modified Eagle's medium (DMEM) (11995065), along with Penicillin-Streptomycin (P/S) (10,000 U/mL) (15140122) and 0.25% trypsin-EDTA (25200056). All reagents, including DMEM, P/S, and trypsin-EDTA, were obtained from this supplier. Fetal bovine serum (FBS) (A5669701) and Horse serum (16050122) were sourced from GIBCO BRL (Grand Island, NY, USA). QUANTI-Blue™ solution (rep-qbs) and CpG2006 (tlrl-2006) were sourced from InvivoGen (San Diego, CA, U.S.A). H&E staining kit (G1120), Artificial Saliva pH 6.0 (A7994) and Artificial Saliva pH 7.0 (A7991) were sourced from Solarbio (Beijing, China). Paraformaldehyde (4%) (BL539A) was sourced from Biosharp (Hefei, China). Quant-iT PicoGreen dsDNA assay kit (P11495), TRIzol (15596018), calf thymus DNA (15633019), anti-TLR9 (MA5-38645), anti-TNF-a (RM9011), anti-IL-6 (701028), anti-p53 (MA5-12557), ELISA kit for human TNF-α and IL-6 (KAC1751, BMS213-2) and An ELISA kit for detecting cytokines mouse TNF-α and IL-6 (BMS607-3, BMS603-2) were obtained from Thermo Scientific, located in Waltham, Massachusetts, USA. These reagents, including TNF-α and IL-6, were procured from this supplier for the study. The Anti-Ki67 antibody (ab15580) was sourced from Abcam (Cambridge, UK). The Anti-Rabbit HRP-DAB IHC Detection Kit (catalog #CTS005) was sourced from Novus, a biotechnology company headquartered in Minneapolis, MN, USA. The PrimeScript™ FAST RT Reagent Kit, which includes a gDNA Eraser (RR092A), along with TB Green® Premix Ex Taq™ (Tli RNaseH Plus) (RR420A), was acquired from Takara Bio, based in Beijing, China. These reagents were sourced for the study. DNeasy Blood & Tissue Kit (69504) was purchased from QIAGEN (Hilden, Germany). Cell Counting Kit-8 (96992), Goat serum (S26F-MLSG), branched polyethylenimine (PEI, Mw 25 kDa) (408727), ammonium nitrate (1.01187), tetraethyl orthosilicate (8.00658), (3-glycidyloxypropyl)trimethoxysilane (440167), bis[3-(triethoxysilyl)propyl]diselenide (15200), triethanolamine (8.22341), cetyltrimethylammonium tosylate (CTAT) (8.14692), and 4-Nitroquinoline-1-Oxide (N8141) was sourced from Sigma-Aldrich, based in St. Louis, MO, USA. All supplementary reagents were sourced from commercial sources and utilized without further purification.

Synthesis of MSN and MSN-PEI

MSN-PEI were prepared as our protocol reported previously[25]. To begin with, MSN

was synthesized. A mixture containing 1.2 g of cetyltrimethylammonium tosylate (CTAT) and 0.3 g of triethanolamine was prepared by dissolving the components in 80 mL of deionized water. The solution was then stirred at 80°C for one hour. After preparing the surfactant solution, a separate solution comprising 4.0 g of bis[3-(triethoxysilyl)propyl]diselenide (BTESePD) and 6.0 g of tetraethyl orthosilicate was slowly added dropwise. The resulting mixture was stirred vigorously at 80°C for 4 hours at a speed of 1000 rpm. The reaction products were then isolated through centrifugation. The collected material was washed multiple times with ethanol and then refluxed in an ethanol solution containing 1% w/v ammonium nitrate for 12 hours. Following this, the diselenide-bridged MSN was purified, washed again, and refluxed in ethanol to remove CTAT. To synthesize MSN-PEI, 1.0 g of MSN was dispersed in 250 mL of toluene under ultrasonic treatment. Then, 1.5 mL of (3-glycidyloxypropyl)trimethoxysilane was added, and the mixture was refluxed at 80°C for 24 hours, yielding epoxy silane-functionalized MSN. After purification, 500 mg of the functionalized MSN was dispersed in 250 mL of a PEI 25K solution (1 mg/mL) and stirred at room temperature for 24 hours. Finally, the diselenide-bridged MSN-PEI was collected, thoroughly washed, and dried for further use.

Characterization of MSN-PEI

Transmission electron microscope (JEOL, Ltd., Japan) and scanning electron microscope (FEI Quanta 200F) were used to observe the morphology of MSN and MSN-PEI. The hydrodynamic diameter and zeta potential of MSN and MSN-PEI were measured with a Zetasizer (Nano ZS90, Malvern Panalytical). Barrett-Joyner-Halenda (BJH) and Brunauer-Emmett-Teller (BET) methods were used to measure the pore size distribution and specific surface area. The PEI content of MSN-PEI was determined by thermogravimetric analysis (TGA, PerkinElmer, U.S.A.). Degradation of MSN-PEI (100 μg/mL) in 100 μM of H2O2 solution at 37°C under constant rotation was evaluated, with samples collected for TEM analysis at 24h and 48h.

Stability of MSN-PEI

MSN-PEI was collected by centrifugation and the supernatant was discarded. The pellet was resuspended in artificial saliva at pH 6.0 or pH 7.0 to a final concentration of 1 mg/mL and incubated at 37°C with shaking at 100 rpm. At 1, 3, 6, 12 and 24 h, 100 μL of the suspension was collected and diluted with 900 μL deionized water prior to analysis. The hydrodynamic particle size and zeta potential were measured using a Zetasizer. For morphology assessment, samples collected at 24 h were deposited onto copper grids and examined by transmission electron microscopy.

Instrumentation

A JEOL, Ltd. (Japan) transmission electron microscope (TEM) and an FEI Quanta 200F scanning electron microscope (SEM) were utilized to investigate the morphological characteristics of MSN and MSN-PEI. The Zetasizer (Nano ZS90, Malvern Panalytical) was used to determine the zeta potential and hydrodynamic diameter of both MSN-PEI and MSN. The PEI content in MSN-PEI was quantified via thermogravimetric analysis (TGA) using a PerkinElmer system (USA).

Cells

The American Type Culture Collection (ATCC), located in Manassas, VA, USA, provided RAW 264.7 cells (catalog #TIB-71) and CAL 27 cells (catalog #CRL-2095). Both cell lines, RAW 264.7 and CAL 27, were sourced from this repository. HEK-Blue TLR9 reporter cells (catalog #hkb-htlr9) were acquired from InvivoGen, headquartered in San Diego, CA, USA. RAW 264.7 cells were cultured in DMEM supplemented with 10% fetal bovine serum (FBS), 1 mM sodium pyruvate, and 1% penicillin-streptomycin, maintained at 37°C under a humidified environment with 5% CO_2. In the same manner, CAL 27 cells were grown in DMEM enriched with 10% FBS, 4.5 g/L glucose, and 4 mM L-glutamine, 1.5 g/L $NaHCO_3$, 1 mM sodium pyruvate, and 1% penicillin-streptomycin under equivalent environmental conditions. HEK-Blue TLR9 reporter cells were propagated in DMEM containing 10% FBS and 1% penicillin-streptomycin, incubated at 37°C under a 5% CO_2 atmosphere.

Extraction and quantification of cfDNA

cfDNA was extracted from saliva or serum using the DNeasy Blood & Tissue Kit (QIAGEN, Germany). The levels of cfDNA present in saliva, plasma, and interstitial fluid were quantified with the Quant-iT PicoGreen dsDNA Assay Kit.

Enzyme-linked immunosorbent assay (ELISA)

Saliva, plasma, and interstitial fluid were prepared as described above. TNF-α and IL-6 were measured in the supernatants using the ELISA kits according to the manufacturer's protocol.

Mechanical sensitivity (Von Frey) test

Mechanical hypersensitivity was evaluated by determining paw withdrawal thresholds in reaction to von Frey filaments (Schriesheim, Germany). Following a 5-minute

habituation period in a plastic enclosure placed on a mesh platform, a calibrated monofilament was applied perpendicularly to the midsole of the plantar surface of the hind paw. The filament was gently pressed upward and held in place for approximately 3 seconds, using Dixon's up-down method. Responses like quick paw withdrawal, paw licking, or shaking were documented. The force needed to elicit a withdrawal response in half of the subjects (median withdrawal threshold) was calculated using Dixon's statistical method, following previously described procedures.[1]

Statistical analysis

The two-tailed Student's *t*-test was used to compare the mean values of the two groups. A one-way analysis of variance (ANOVA) and Tukey's multiple comparisons test was used for multiple groups. P values of <0.05 were regarded as indicating statistically significant differences. All experiments were conducted at least twice with similar results. A representative result is shown in the paper. Statistical analyses were conducted with GraphPad Prism 9. Quantitative analyses of Olympus VS200 microscopy images were carried out with ImageJ.

References

1 Lu, F. *et al.* Objective and Quantitative Evaluation of Spontaneous Pain-Like Behaviors Using Dynamic Weight-Bearing System in Mouse Models of Postsurgical Pain. *J Pain Res* **15**, 1601-1612 (2022). https://doi.org:10.2147/jpr.S359220

Supplementary Tables

Table S1. Clinicopathologic characteristics of patients with OSCC, including age, sex, tumor site and TNM stage.

Patient number	Site of OSCC	Sample collection	TNM
1	Left buccal mucosa	Blood, saliva, tissue	T2N0M0
2	Right buccal mucosa	Blood, saliva, tissue	T4aN2bM0
3	Right buccal mucosa	Blood, saliva, tissue	T3N2bM0
4	Right buccal mucosa	Blood, saliva, tissue	T2N1M0
5	Right buccal mucosa	Blood, saliva, tissue	T1N0M0
6	Right buccal mucosa	Blood, saliva, tissue	T3N0M0
7	Left buccal mucosa	Blood, saliva, tissue	T2N0M0
8	Left tongue	Blood, saliva, tissue	T1N0M0
9	Left tongue	Blood, saliva, tissue	T2N0M0
10	Left tongue	Blood, saliva, tissue	T1N0M0
11	Left tongue	Blood, saliva, tissue	T1N1M0
12	Right tongue	Blood, saliva, tissue	T1N1M0
13	Right tongue	Blood, saliva	T2N0M0
14	Right tongue	Blood, saliva	T2N2aM0
15	Right tongue	Blood, saliva	T1N0M0
16	Right tongue	Blood, saliva, tissue	T3N2bM0
17	Floor of the mouth	Blood, saliva, tissue	T1N0M0
18	Floor of the mouth	Blood, saliva, tissue	T2N1M0
19	Floor of the mouth	Blood, saliva, tissue	T3N2bM0
20	Left palate	Blood, saliva, tissue	T1N0M0
21	Left lower lip	Blood, saliva, tissue	T1N0M0
22	Lower lip center	Blood, saliva, tissue	T1N1M0
23	Left lower lip	Blood, saliva, tissue	T1N0M0

Tumor sites are recorded as tongue, lip, buccal mucosa, floor of the mouth and palate; all patients had no documented distant metastasis (M0).

Table S2. Primers for quantitative real-time PCR.

Primer	Forward	Reverse
Gapdh	GAAGGTGAAGGTCGGAGTC	GAAGATGGTGATGGGATTT
Tnf-α	GGCTCCAGGCGGTGCTTG	CAGATAGATGGGCTCATACCA
Arg-1	GCAGAAGTCAAGAAGAACGG	GGTTGTCAGTGGAGTGTTG
Cd206	GAGCAAACATACCTGACAGGATTA	GGACTTCCTGGTAACCAGTTCA
Cd86	CCATCAGCTTGTCTGTTTCATTCC	GCTGTAATCCAAGGAATGTGGTC
Il6	GGTGTTGCCTGCTGCCTTCC	GTTCTGAAGAGGTGAGTGGCTGTC
Cd163	CTTAAATGTGGAGTTGCCCTT	ACATAATGAAGCACCTAGAGC
Tlr9	GGGACCTCGAGTGTGAAGCA	CTGGAGCTCACAGGGTAGGAA
Sox2	GTGAGCGCCCTGCAGTACAA	GCGAGTAGGACATGCTGTAGGTG
Oct4	GCTGGATGTCAGGGCTCTTTG	TTCAAGAGATTTATCGAGCACCTTC
Cd86	TGCTCATCTATACACGGTTACC	TGCATAACACCATCATACTCGA
Cd163	ATCAACCCTGCATCTTTAGACA	CTTGTTGTCACATGTGATCCAG
Cd204	GGACACTGATAGCTGCTCCGAATC	CACGAGGAGGTAAAGGGCAATCAG
Arg1	GGACCTGCCCTTTGCTGACATC	TCTTCTTGACTTCTGCCACCTTGC

Supplementary Figures

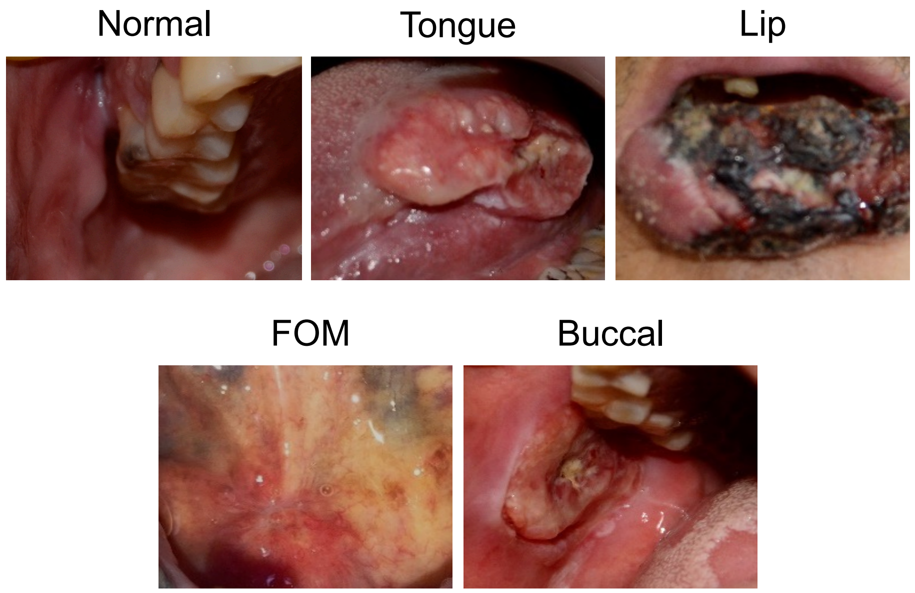

Figure S1. Representative intraoral photographs of normal oral mucosa and OSCC lesions at different sites, including tongue, lip, buccal mucosa and floor of the mouth. The anatomical site of each panel is indicated by the text label above the image. In the normal, tongue and buccal mucosa photographs, the dentition provides additional landmarks for orientation, whereas the floor-of-the-mouth lesion is shown at the junction between the lingual frenulum and the adjacent floor-of-mouth mucosa.

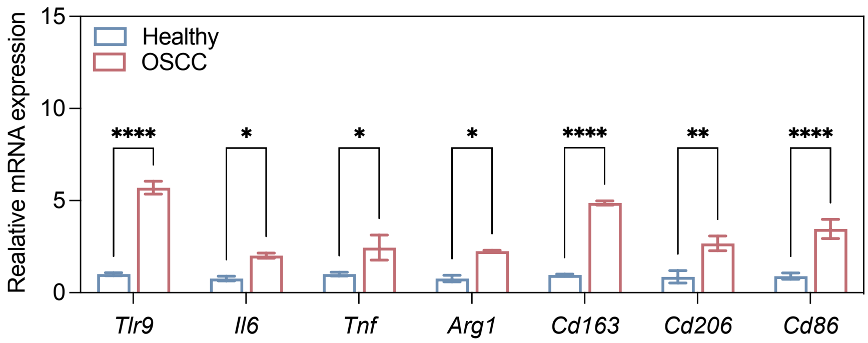

Figure S2. mRNA expression levels of *Tlr9*, *Il6*, *Tnf*, *Arg1*, *Cd163*, *Cd206*, and *Cd86* in RAW 264.7 cells by plasma from healthy volunteers and patients with OSCC. (n = 3 per group; Data are presented as mean ± SEM; *$P < 0.05$, **$P < 0.01$, ****P < 0.0001 by two-tailed Student's *t*-test. *$P < 0.05$, **$P < 0.01$, ****$P < 0.0001$).

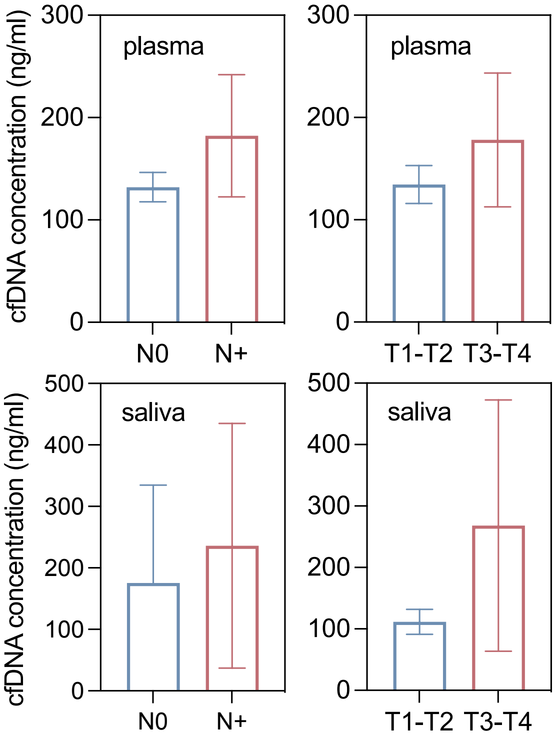

Figure S3. cfDNA levels in saliva and plasma from patients with OSCC and lymph node metastasis. (n = 3 per group; Data are presented as mean ± SEM). N0, negative lymph node metastasis; N+, positive lymph node metastasis; T1, the primary lesion's diameter is smaller than or equal to 3 cm; T2, the primary lesion's diameter is larger than 3 cm but smaller than or equal to 5 cm; T3, the primary lesion's diameter is larger than 5cm but smaller than or equal to 7cm; T4, the primary lesion's diameter is larger than 7cm.

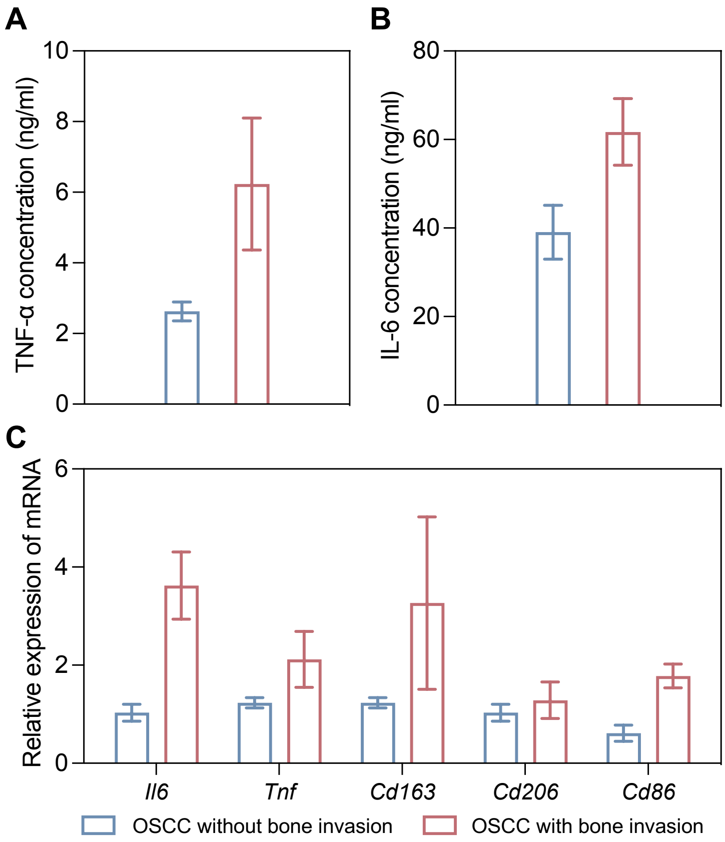

Figure S4. (**A**) TNF-a and (**B**) IL-6 levels, and (**C**) mRNA expression levels of *Il6*, *Tnf*, *Cd163*, *Cd206*, and *Cd86* in bone tissue of healthy volunteers and patients with OSCC (n = 3. Data are means ± SEM).

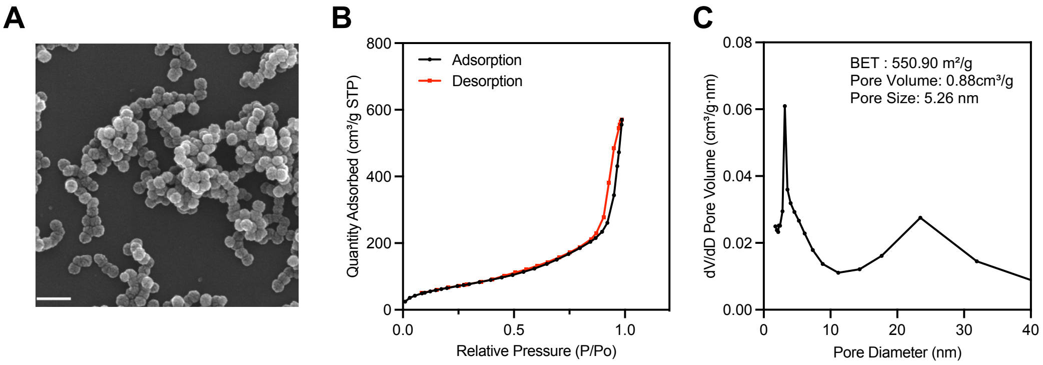

Figure S5. (**A**) Representative SEM image of MSN. (Scale bar, 200 nm.) (**B**) Nitrogen adsorption desorption isotherm and (**C**) pore size distribution of MSN.

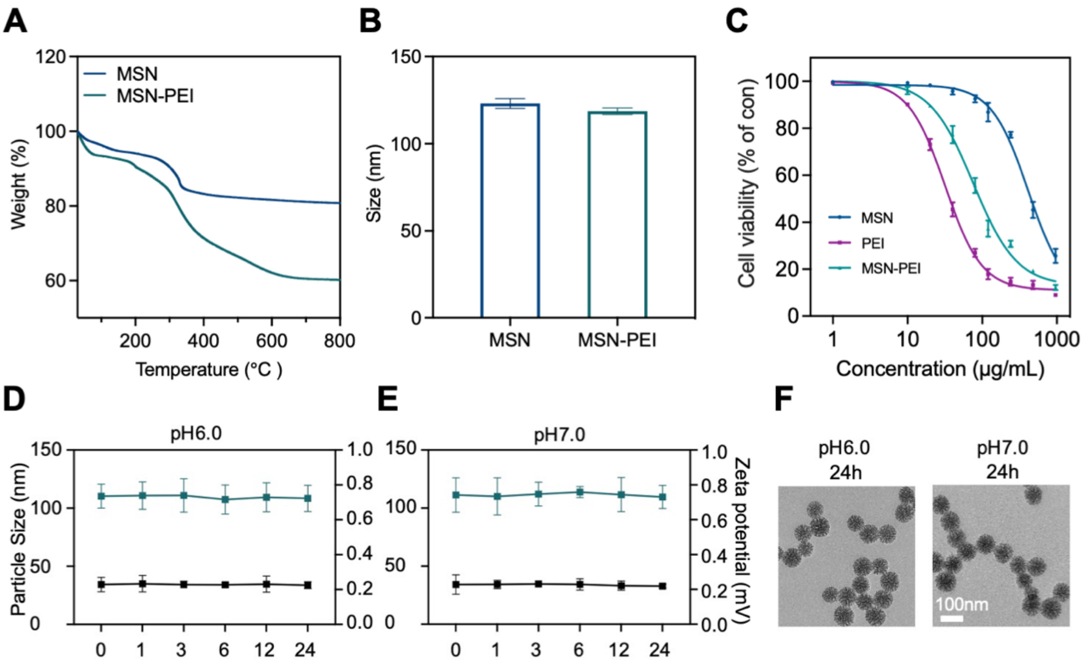

Figure S6. (**A**) The thermal decomposition rate of the MSN changes after conjugating PEI. (**B**) Size of MSN and MSN-PEI. (n = 3 per group. Data are presented as mean ± SD.) (**C**) Relative fluorescence intensity of CCK-8 in HEK-TLR9 reporter cells after 24 hours incubation with different with various concentrations of MSN, PEI, and MSN-PEI. (**D**) Hydrodynamic particle size and zeta potential of MSN-PEI incubated at pH 6.0 over 24 h, measured at 0, 1, 3, 6, 12 and 24 h. (**E**) Hydrodynamic particle size and zeta potential of MSN-PEI incubated at pH 7.0 over 24 h, measured at 0, 1, 3, 6, 12 and 24 h. (**F**) Representative TEM images of MSN-PEI after 24 h incubation at pH 6.0 and pH 7.0. Scale bar, 100 nm. (n = 3 per group. Data are presented as mean ± SEM).

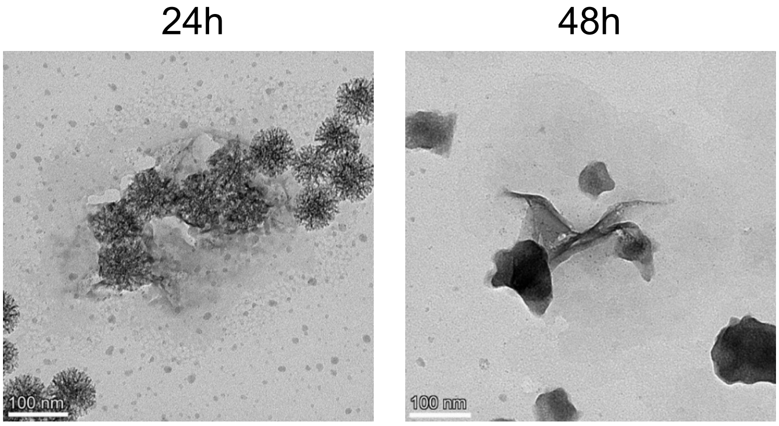

Figure S7. Representative TEM images of MSN-PEI after 24h and 48h incubation in H_2O_2. (Scale bar, 100 nm.)

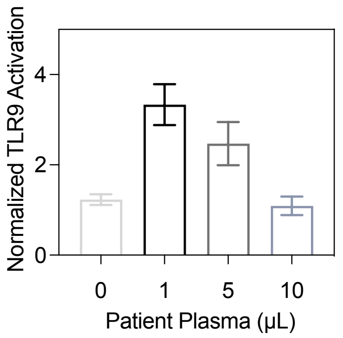

Figure S8. Activation of HEK-TLR9 reporter cells by adding different volumes of OSCC patient plasma in 200 μl medium system for 24 hours. The corresponding SEAP activity in supernatants from each group is determined with a QUANTI-Blue assay at OD620. (n = 3 per group.)

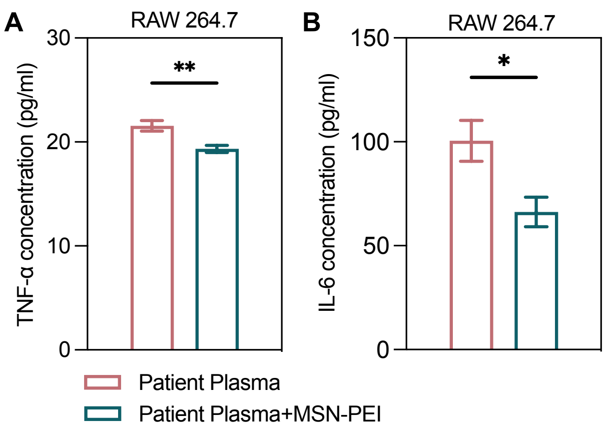

Figure S9. Inhibitory effects of MSN-PEI on patient plasma-induced TNF-α and IL-6 secretion by RAW264.7 cells. (n = 3 per group. Data are presented as mean ± SEM. *$P < 0.05$, **$P < 0.01$ by two-tailed Student's *t*-test.)

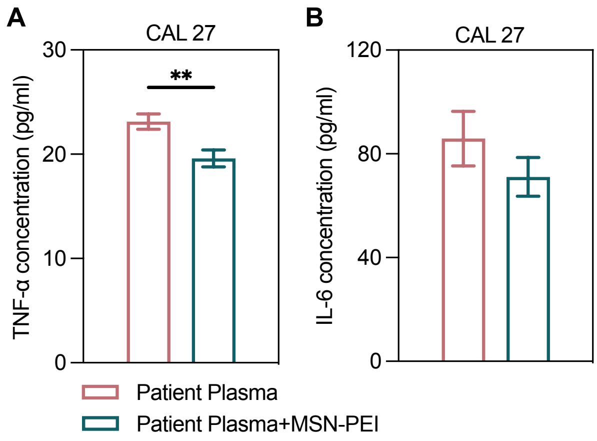

Figure S10. Inhibitory effects of MSN-PEI on patient plasma-induced TNF-α and IL-6 secretion by CAL 27 cells. (n = 3 per group. Data are presented as mean ± SEM. $^{**}P < 0.01$ by two-tailed Student's *t*-test.)

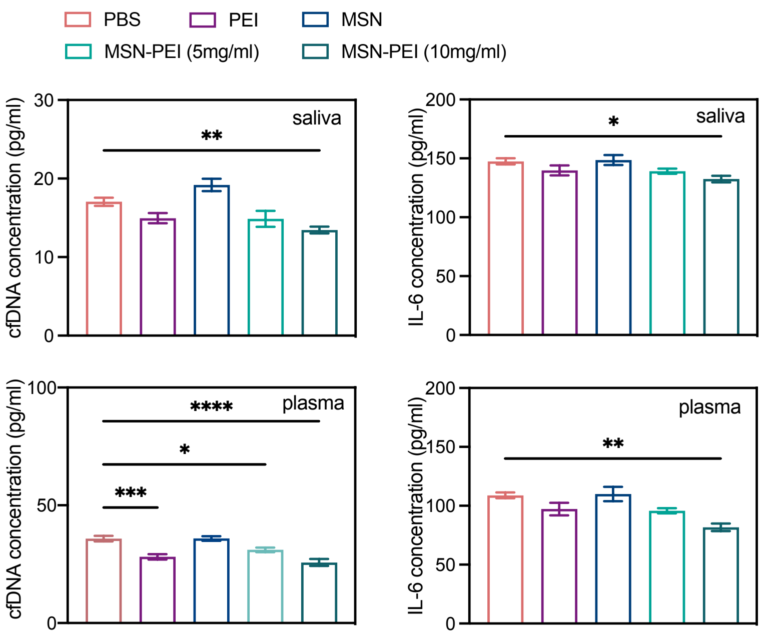

Figure S11. cfDNA and IL-6 levels in saliva and plasma of mice with 4-weeks-4NQO stimuli with different treatment (PBS, 1mg/mL PEI, 10 mg/mL MSN, and 10 mg/mL MSN-PEI).

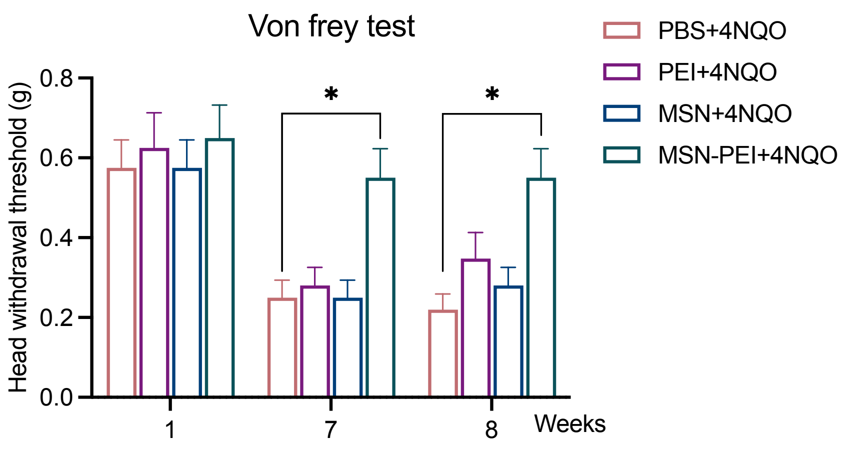

Figure S12. Von frey test of mice from different groups after 8-weeks-4NQO stimuli.

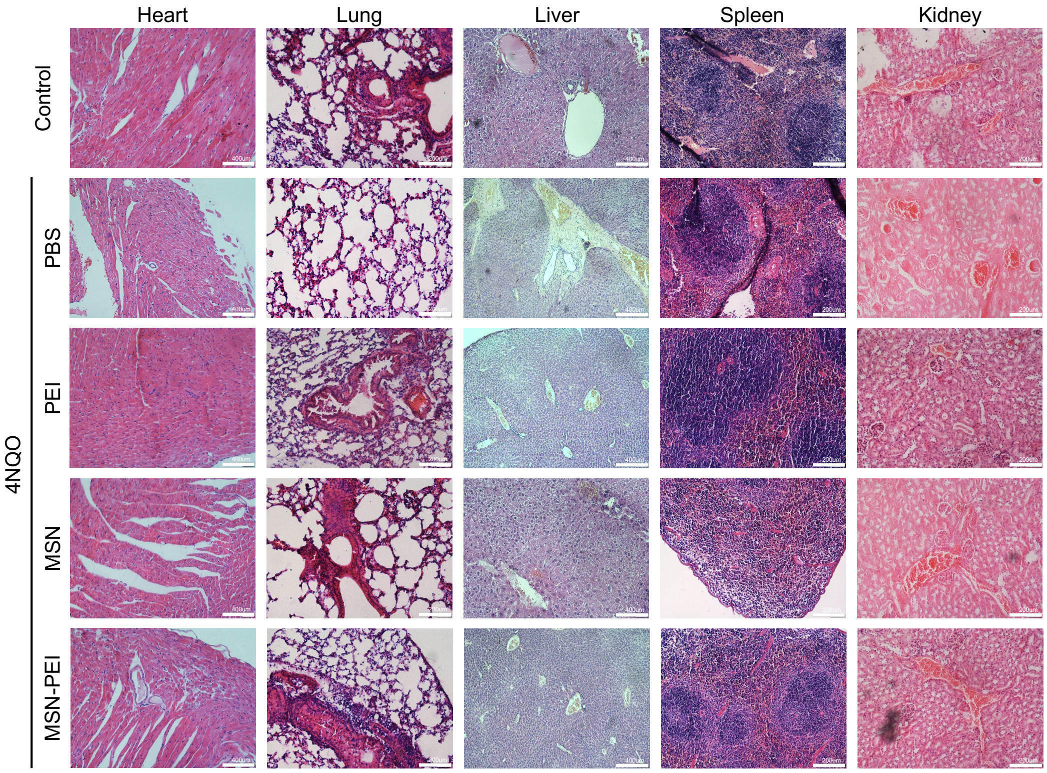

Figure S13. H&E staining of the heart, liver, spleen, lung, and kidney of mice from different groups after 8-weeks-4NQO stimuli. (Scale bar, 400 μm or 200 μm, respectively.)
